# Aspirin for Primary Stroke Prevention; Evidence for a Differential Effect in Men and Women

**DOI:** 10.3389/fneur.2022.856239

**Published:** 2022-06-21

**Authors:** Zuzana Gdovinova, Christine Kremer, Svetlana Lorenzano, Jesse Dawson, Avtar Lal, Valeria Caso

**Affiliations:** ^1^Neurology Department, Faculty of Medicine P.J. Safarik University Košice, L. Pasteur University Hospital, Košice, Slovakia; ^2^Neurology Department, Skåne University Hospital, Department of Clinical Sciences Lund University, Malmö, Sweden; ^3^Department of Human Neurosciences, Sapienza University of Rome, Rome, Italy; ^4^College of Medical, Veterinary & Life Sciences, Institute of Cardiovascular and Medical Sciences, University of Glasgow, Glasgow, United Kingdom; ^5^European Stroke Organisation (ESO), Basel, Switzerland; ^6^Stroke Unit, Santa Maria della Misericordia Hospital, University of Perugia, Perugia, Italy

**Keywords:** aspirin, primary prevention, ischemic stroke, hemorrhagic stroke, men, women

## Abstract

**Background:**

The use of aspirin for primary prevention of cardiovascular events in men and women remains controversial. Our study aimed to investigate the role of aspirin in primary stroke prevention in men and women and the effect of aspirin on risk of ischemic stroke in patients with covert cerebral small vessel disease (ccSVD).

**Methods:**

We performed systematic searches of the PubMed, and Cochrane Library databases, covering the period from the inception of each database to May 2021. The incidence of any ischemic stroke (IS) or hemorrhagic stroke (HS) was the main outcome. The incidence of stroke overall, both ischemic (IS) and hemorrhagic (HS), was the main outcome.

**Results:**

From 531 abstracts, 11 randomized control trials which assessed primary prevention of cardiovascular events in men and women were included. Only one study assessed the risk of aspirin in people with ccSVD. In women, there was significant decrease in the risk of stroke (OR 0.85 [95% CI 0.73, 0.99], *p* = 0.03) and IS (OR 0.76 [0.63, 0.93], *p* = 0.008) with aspirin compared to placebo while no increase in the risk of HS was found (OR 1.78 [0.61, 5.19], *p* = 0.29). In men, aspirin did not affect the risk of stroke (OR 1.13 [0.97, 1.31], *p* = 0.12) and IS (OR 0.94 [0.67, 1.32], *p* = 0.72) but increased the risk of HS with borderline statistical significance (OR 1.99 [0.99, 4.03], *p* = 0.05) compared to placebo. Aspirin significantly increased major bleedings in both sexes (*p* < 0.05). We found no evidence to support the use of aspirin in patients with ccSVD.

**Conclusion:**

Our meta-analysis suggests aspirin is effective in primary prevention of stroke and IS in women with no clear increased risk of HS. However, it was associated with an overall increased risk of bleeding. Aspirin is not recommended in ccSVD.

## Introduction

The use of aspirin for the primary prevention of cardiovascular events in men and women remains controversial (1–7). The antithrombotic effect of aspirin is primarily related to the irreversible inhibition of the enzyme cyclooxygenase in platelets resulting in a decreased production of prostaglandins and thromboxane A2. Furthermore, aspirin reduces inflammation by forming nitric oxide radicals and protects endothelial cells from oxidative stress. Sex hormones are known to have differential effects on platelet function, with testosterone promoting platelet activity and estrogen inhibiting (8–10).

Based on these premises, our study aimed to investigate the role of aspirin in primary prevention in men and women. In addition, because the risk benefit ratio of antiplatelets may differ in people with cerebral small vessel disease, we also explored the effect of aspirin on risk of stroke risk in people with covert cerebral small vessel disease (ccSVD). Covert small vessel disease was defined as: Cerebral small vessel disease (SVD) with the presence of brain lesions found on CT or MR brain imaging or pathology examination, thought to have resulted from disease of the small blood vessels that perforate into the brain, primarily affecting the white matter and deep gray matter. The full spectrum includes covert cerebral SVD (ccSVD) detected incidentally on neuroimaging, and SVD-related clinical presentation with stroke, cognitive decline or dementia, mood or physical dysfunction (11).

We performed systematic searches of the PubMed, and Cochrane Library databases, covering the period from the inception of each database to May 2021; the incidence of stroke, both ischemic (IS) and hemorrhagic (HS), was the main outcome.

## Methods

### Literature Search and Study Selection

A comprehensive search of Medline, EMBASE, CINAHL, SCOPUS was performed. The search terms aspirin, stroke, women and their corresponding Medical Subject Heading (MeSH) terms were used. The search strategy was (((Aspirin OR antiplatelet^*^ OR dual antiplatelet therapy OR DAPT) AND (stroke OR TIA OR CVA OR cerebrovascular accident OR cerebrovascular apoplexy OR transient ischemic attack OR cerebrovascular infarct^*^ OR cerebrovascular embolism OR brain ischaemia OR wind stroke OR cerebral embolism OR brain infarct^*^ OR intracranial hemorrhages) AND (women OR females OR men OR male OR sex difference OR gender difference OR sex factor^*^ OR gender factor^*^)).ti,ab.). The exclusion criteria were as follows: (i) people without stroke; (ii) Intervention: no aspirin, no antiplatelets therapy; (iii) Outcome: non-relevant outcomes; (iv) Study designs: narrative reviews, letter to editor, case report, commentary, or editorial.

**Figure 1:**
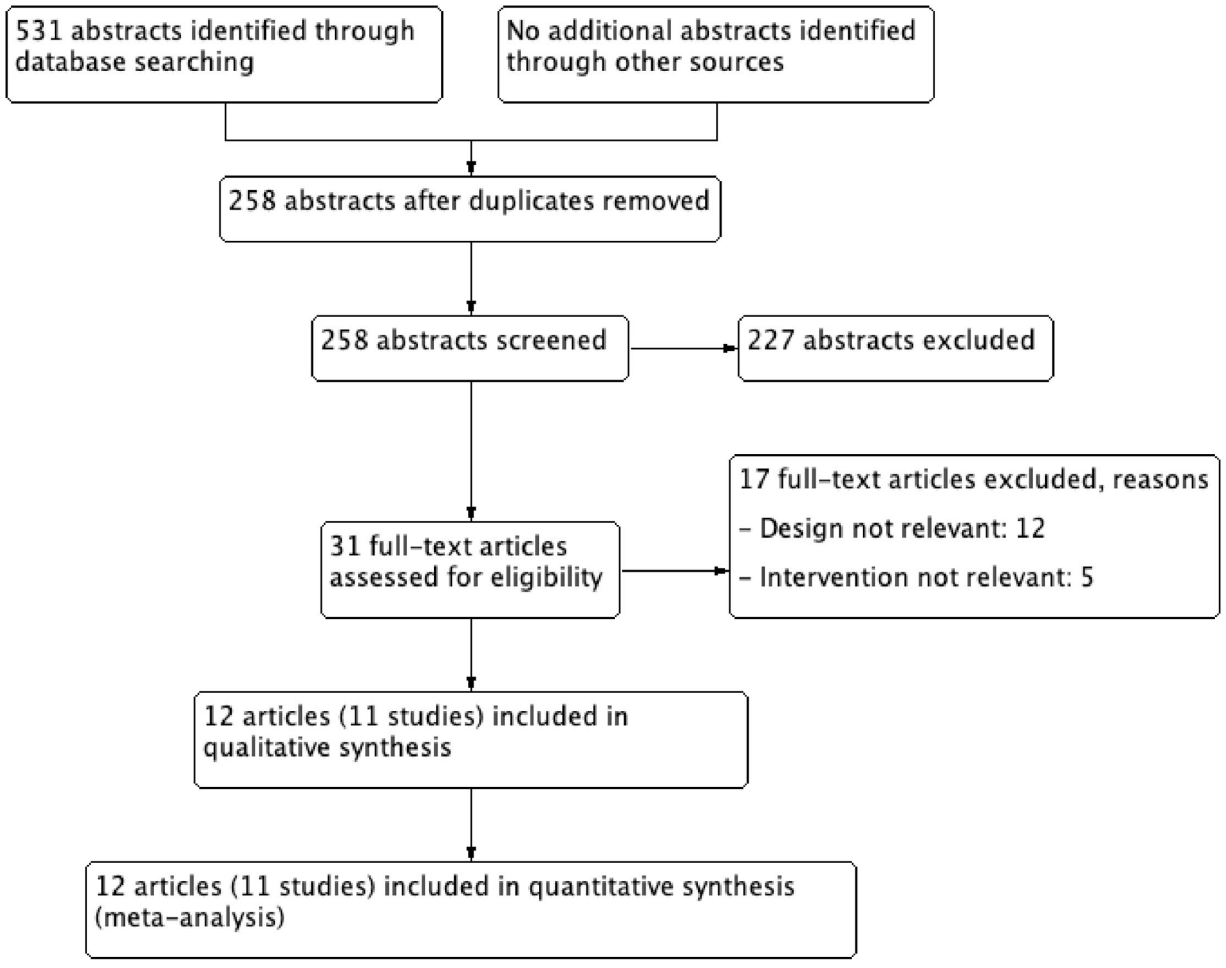
Study flow chart. A total 531 abstracts identified through database searching (PubMed, and Cochrane Library databases), finally 11 studies (12 articles) were included in quantitative synthesis (meta-analysis).

A total of 531 abstracts were identified from different searches and uploaded on the COVIDENCE software (Covidence systematic review software, Veritas Health Innovation, Melbourne, Australia). This systematic review and meta-analysis were conducted by following the guidelines of PRISMA (12). After removing the duplicates, 258 abstracts were screened by title and abstract. Of these, 31 articles were selected for full text screening and 12 articles (11 studies) published before April 2020 were selected for data extraction and meta-analysis (Figure 1 and Table 1). The selection of the abstracts and articles were performed independently and in duplicate. The data extraction was done by one person and checked by others. The incidence of stroke, both ischemic and hemorrhagic, was considered the primary outcome. Secondary outcomes were ischemic stroke, hemorrhagic stroke, and bleeding episodes.

**Table 1:** Randomized control trials included in the meta-analysis.

**Studies**	**Included participants**
WHS	Asymptomatic women, 45 years of age or older
AAA	Patients with a low ankle brachial index (ABI) indicating atherosclerosis, aged 55–75 years
ETDRS	Patients with DM 1 and DM 2 and aged 18–70 years
JPAD	Patients with DM 2
POPADAD	Patients with DM and asymptomatic peripheral arterial disease, aged 40 or more years, aspirin was combined with antioxidants
JPPP	Patients aged 60 to 85 years with DM, hypertension and dyslipidemia
HOT	Patients allocated to a target blood pressure level and randomly assigned to aspirin or placebo group
PPP	Vitamin E was added to low dose of aspirin in patients with hypertension, hypercholesterolemia, diabetes, obesity, family history of premature myocardial infarction, or individuals who were elderly
ASCEND	Patients ≥ 40 years old with DM1 and DM2 without ASCVD, randomised to aspirin 100 mg/D or placebo
ASPREE^*^	Patients ≥ 70 years old (≥ 65 years old for Hispanic and African American patients) without life-limiting chronic illness, dementia, physical disability or documented cardiovascular or cerebrovascular disease, randomised to aspirin 100 mg/D or placebo
ARRIVE	Men ≥ 55 years and 2–4 risk factors, women aged ≥ 60 years and with ≥ 3 risk factors

*WHS, Women's Health Study; AAA, Aspirin for Asymptomatic Atherosclerosis; ETDRS, Early Treatment Diabetic Retinopathy Study; JPAD, Japanese Primary Prevention of Atherosclerosis With Aspirin for Diabetes; POPADAD, The prevention of progression of arterial disease and diabetes; JPPP, The Japanese Primary Prevention Project; HOT, Hypertension Optimal Treatment Trial; PPP, Primary Prevention Project; ASCEND, A Study of Cardiovascular Events in Diabetes; ASPREE, Aspirin in Reducing Events in the Elderly; ARRIVE, Use of Aspirin to Reduce Risk of Initial Vascular Events in patients at moderate risk of cardiovascular disease.*

* *results published in 2 articles*.

### Data Analysis and Statistical Methods

Meta-analysis was performed using Review Manager (RevMan) 5.3 COCHRANE Collaboration software when more than one study reported the outcome and number of subjects were ≥ 6 in each group. Odds ratio (OR) and 95% confidence intervals (CI) for dichotomous variables were calculated. *I*^2^ statistic, an expression of inconsistency of studies' results and describing the percentage of variation across studies due to heterogeneity rather than by chance, was calculated. A high value of *I*^2^ (>50%) and *p*-value <0.05 indicate statistically significant heterogeneity among the studies for an outcome. The reasons for high heterogeneity were explored. A random effects model was used for all outcomes. The publication bias was assessed by looking at the asymmetry of a funnel plot. The funnel plot was generated if at least 10 studies were included in a meta-analysis.

## Results

From 531 abstracts, 31 articles were selected for full text screening and of these, 12 met the eligibility criteria and contained the results of 11 randomized control trials (RCTs) (Table 1) (13–24). The flowchart of the study is shown in Figure 1. In all included studies except for WHS, both men and women were enrolled (representation of women was 29.5–70%) and heterogenous populations were studied. Three of the RCT compared ASA with placebo and were published in four articles 2018 (21–24).

Another study sought to detect if aspirin reduces the risk of ischemic stroke or increases hemorrhagic stroke risk in patients with covert cerebral small vessel disease (25). One RCT included patients ≥ 45 years with at least one silent brain infarct (SBI) but no previous clinical cerebrovascular events for randomization to aspirin 100 mg or placebo. The primary endpoint was the combined endpoint of ischemic stroke, TIA, and new silent brain infarcts detected by MRI (25).

### RCTs on Aspirin as the Primary Prevention of Stroke

**The WHS (Women's Health Study) trial** was the only trial which included only women. In this trial 100 mg of aspirin on alternate days or placebo was prescribed to 39 876 initially asymptomatic women 45 years of age or older, who were followed up for 10 years for a first major vascular event (non-fatal MI, non-fatal stroke, or cardiovascular death). Although there was a non-significant 9% reduction (RR, 0.91; 95% CI, 0.80–1.03; *P* = 0.13) in the combined primary endpoint among women, the study found a statistically significant 17% reduction in the risk of stroke (RR, 0.83; 95% CI, 0.69–0.99; *P* = 0.04) There was a 24% reduction in the risk of ischemic stroke (RR, 0.76; 95% CI, 0.63–0.93; *P* = 0.009) and a non-significant increase in the risk of hemorrhagic stroke (RR, 1.24; 95% CI, 0.82–1.87; *P* = 0.31). Aspirin therapy was associated with a 22% reduction in the risk of transient ischemic attack (RR, 0.78; 95% CI, 0.64–0.94; *p* = 0.01).

Occurrence of gastrointestinal hemorrhage requiring transfusion, was more frequent in the aspirin group (RR, 1.40; 95% CI, 1.07–1.83; *P* = 0.02). The most consistent benefit for aspirin was in women ≥ 65 years of age at study entry, among whom the risk of major cardiovascular events was reduced by 26% (RR, 0.74; 95% CI, 0.59–0.92; *P* = 0.008), including a 30% reduction in the risk of ischemic stroke (RR, 0.70; 95% CI, 0.49–1.00; *P* = 0.05). However, there was no significant benefit when the combination of IS and hemorrhagic stroke was considered (RR, 0.78; 95% CI, 0.57–1.08; *P* = 0.13). Based on the WHS results, aspirin is recommended for primary prevention for women after consideration of the 10-year risk of cerebrovascular disease (CVD) and of whether this and age outweigh the risk of hemorrhage (13).

**In the AAA (Aspirin for Asymptomatic Atherosclerosis) trial** 100 mg aspirin once daily was compared with placebo in men and women free of clinical cardiovascular disease and with low ( ≤0.95) Ankle Brachial Index (ABI). Approximately, 70% of trial participants (3, 350) were women. Participants were followed up for a mean (SD) of 8.2 (1.6) years A primary endpoint event (fatal or non-fatal coronary event or stroke or revascularisation) occurred in 357 participants (13.5 per 1,000 person/years, 95% CI, 12.2–15.0) and no statistically significant difference was found in event rates over time between the groups (aspirin, 13.7; 95% CI, 11.8–15.9 vs. placebo, 13.3; 95% CI, 11.4–15.4 events per 1 000 person/years; hazard ratio [HR], 1.03; 95%CI, 0.84–1.27) There was no difference between the groups also for secondary endpoints: all initial vascular events defined as a composite of a primary endpoint event or angina, intermittent claudication, or transient ischemic attack and all-cause mortality. The comparison of the primary endpoint by sex, age, and ABI, found lower event rates in women than in men, but in both groups, the difference was higher in aspirin group (men, event rate in aspirin group 27.4 [22.2–33.5)] vs. placebo group 23.9 [19.0–29.6], 95% CI 1.15 [0.86–1.54]; women in aspirin group 8.8 [7.0–10.8] vs. placebo group 9.6 [7.7–11.7], 95% CI 0.92 [0.68–1.23]) and in patients < 62 years of age. Although no statistically significant effect of aspirin on major events was found, the HR and 95% CIs did not rule out the possibility of a risk reduction of up to 16% (or an increased risk up to 27%). To achieve this, it means that 500–600 people from the general population would need to be screened and prescribed aspirin to prevent a single major cardiovascular event over an 8-year period. Adverse effects such as major hemorrhage, gastrointestinal ulcer and fatal intracranial hemorrhage are of particular concern in the context of screening the healthy general population and when the absolute effects of aspirin in reducing major vascular events may be small (14).

**In the ETDRS (Early Treatment Diabetic Retinopathy Study) trial** only patients with diabetes mellitus (DM 1 and DM2), between the ages of 18 and 70 years with different categories of diabetic retinopathy were included, randomly assigned to aspirin 650 mg daily or placebo, and followed up for 5 years. The primary endpoint was mortality from all causes. Among the 3,711 patients enrolled in EDTRS, 1 615 (44%) were women and only a slightly higher relative risk for stroke was reported for females (RR, 1.31; 99% CI, 0.71–2.39) than for males (RR, 1.07; 99% CI, 0.63–1.83) (15).

**In the JPAD (Japanese Primary Prevention of Atherosclerosis with Aspirin for Diabetes) trial** only patients with type 2 diabetes mellitus without history of atherosclerotic disease were enrolled. A low-dose aspirin (81–100 mg) was used, and the median follow-up duration was 4.37 years. The primary endpoint was any atherosclerotic event, which was a composite of sudden death; death from coronary, cerebrovascular, and aortic causes; non-fatal acute myocardial infarction; unstable angina; newly developed exertional angina; non-fatal ischemic and hemorrhagic stroke; transient ischemic attack; or non-fatal aortic and peripheral vascular disease during the follow-up period. Overall, mean (SD) age was 65 (10) years, 44% of 2,539 patients were women. Out of a total of 154 atherosclerotic events, 68 (5.4%) were in the aspirin group and 86 (6.7%) in the non-aspirin group (HR, 0.80; 95%CI, 0.58–1.10; log-rank test, *P* = 0.16) and there was no difference in occurrence of hemorrhagic strokes (6 in the aspirin group, 7 in the non-aspirin group) There were no significant differences between the aspirin group and non-aspirin group in other subgroup analyses, including that by sex (16).

**In the POPADAD (The Prevention Of Progression of Arterial Disease And Diabetes) trial** 1 276 patients (female 56%) with DM1 and DM2 without symptomatic cardiovascular disease aged 40 or more years were included, aspirin 100 mg daily was used with and without antioxidant and compared with placebo alone or with antioxidant. Overall, 233 participants experienced the composite primary endpoint, with an event rate of 2.9 per 100 patient years. No significant differences were found between aspirin and no aspirin in the primary and secondary endpoints In the subgroup analysis, the difference in treatment effect between men and women was not statistically significant (men, event rate in aspirin group 68 [23.8%] vs. placebo group 62 [22.4%], 95% CI 1.04 [0.74–1.47]; women in aspirin group 48 [13.6%] vs. placebo group 55 [15.2%], 95% CI 0.89 [0.60–1.31]) (17).

**In the JPPP (The Japanese Primary Prevention Project) trial** 14 464 patients (women 58%) were aged 60 to 85 years and presenting with DM and also hypertension and dyslipidaemia. Low-dose aspirin (100 mg) once-daily was compared with placebo. The study was terminated prematurely owing to futility; regression analyses indicated that the risk of a primary endpoint event (composite death from cardiovascular causes, non-fatal stroke and non-fatal myocardial infarction) was higher in patients aged 70 years or older vs. those younger than 70 years (parameter estimate, 0.92; HR, 2.51 [95%CI, 2.00–3.14]; *p* < 0.001) and, in terms of sex, in men vs. women (parameter estimate, 0.34; HR, 1.41; 95% CI, 1.14–1.74; *P* = 0.002) (18).

**In the HOT (Hypertension Optimal Treatment Trial) trial** the potential benefit of a low dose of acetylsalicylic acid in the treatment of hypertension was studied. Overall, 18 790 patients (male/female−53%/47%) aged 50–80 years, with hypertension and diastolic blood pressure between 100 and 115 mm Hg (mean 105 mm Hg), were included in the study and randomly assigned to a target diastolic blood pressure. Acetylsalicylic acid 75 mg/day was used in 9 399 patients and 9 391 patients were assigned to placebo. Primary outcomes were major cardiovascular events, defined as all (fatal and non-fatal) myocardial infarctions, all (fatal and non-fatal) strokes, and all other cardiovascular deaths. Aspirin significantly (*p* = 0.03) reduced the major cardiovascular events by 15%, all myocardial infarction was 36% less frequent in the aspirin group with a significant difference (*p* = 0.002). No difference in stroke incidence between patients randomized to acetylsalicylic acid or placebo was observed. However, while fatal bleeds (including cerebral) were equally common in the two groups, non-fatal major bleeds were significantly more frequent among patients receiving aspirin than in those receiving placebo (risk ratio 1.8, *p* < 0.001); minor bleeds were also 1.8 times more frequent among patients who were on aspirin. A specific comparison of the treatment effect in men and women was not made (19).

**In the PPP (Primary Prevention Project) trial** vitamin E 300 mg/day was added to low dose of Aspirin 100 mg/day in patients with hypertension, hypercholesterolemia, diabetes, obesity, family history of premature myocardial infarction, or individuals who were elderly. Slightly more women (57%) out of a total 4 495 patients were included, but a subgroup analysis by sex was not performed. Aspirin lowered the frequency of all the endpoints (major fatal and non-fatal cardiovascular events) being significant for cardiovascular death (from 1.4 to 0.8%; RR, 0.56; 95% CI, 0.31–0.99) and total cardiovascular events (from 8.2 to 6.3%; RR, 0.77; 95% CI, 0.62–0.95). Severe bleedings were more frequent in the aspirin group than in the no-aspirin group (1.1% vs. 0.3%; *p* < 0.0008). Vitamin E showed no effect on any pre-specified endpoint (20).

**In the ASCEND (A Study of Cardiovascular Events in Diabetes) trial** adults who had diabetes but no evident cardiovascular disease were randomly assigned to receive aspirin at a dose of 100 mg daily or matching placebo. The primary efficacy outcome was the first serious vascular event (i.e., myocardial infarction, stroke or transient ischemic attack, or death from any vascular cause, excluding any confirmed intracranial hemorrhage). The primary safety outcome was the first major bleeding event (i.e., intracranial hemorrhage, sight-threatening bleeding event in the eye, gastrointestinal bleeding, or other serious bleeding). Secondary outcomes included gastrointestinal tract cancer. A total of 15 480 participants of at least 40 years of age (female 37.5%) were randomized. During a mean follow-up of 7.4 years, serious vascular events occurred in a significantly lower percentage of participants in the aspirin group than in the placebo group (658 participants [8.5%] vs. 743 [9.6%]; rate ratio, 0.88; 95% confidence interval [CI], 0.79 to 0.97; *P* = 0.01). In contrast, major bleeding events occurred in 314 participants (4.1%) in the aspirin group, as compared with 245 (3.2%) in the placebo group (rate ratio, 1.29; 95% CI, 1.09 to 1.52; *P* = 0.003), with most of the excess being gastrointestinal bleeding and other extracranial bleeding. There was no significant difference between the aspirin group and the placebo group in the incidence of gastrointestinal tract cancer (157 participants [2.0%] and 158 [2.0%], respectively) or all cancers (897 [11.6%] and 887 [11.5%]); long-term follow-up for these outcomes is planned.

Aspirin use prevented serious vascular events in persons who had diabetes and no evident cardiovascular disease at trial entry, but it also caused major bleeding events. The absolute benefits were largely counterbalanced by the bleeding hazard (21).

**In the ASPREE (Aspirin in Reducing Events in the Elderly) trial**, of the 19,114 healthy persons (female 56.4%), who did not have cardiovascular disease, dementia, or disability, of over 70 years of age who were enrolled. Of these, 9 525 were assigned to receive 100 mg of enteric-coated aspirin and 9,589 to receive placebo. The primary composite endpoint was derived from the first endpoint events of death, dementia, and persistent physical disability. A total of 1 052 deaths occurred during a median of 4.7 years of follow-up. The risk of death from any cause was 12.7 events per 1,000 person-years in the aspirin group and 11.1 events per 1 000 person-years in the placebo group (HR, 1.14; 95% CI, 1.01 to 1.29). Cancer was the major contributor to the higher mortality in the aspirin group, accounting for 1.6 excess deaths per 1,000 person-years. Cancer-related death occurred in 3.1% of the participants in the aspirin group and in 2.3% of those in the placebo group (hazard ratio, 1.31; 95% CI, 1.10 to 1.56) (22). The use of low-dose aspirin as a primary prevention strategy in older adults resulted in a significantly higher risk of major hemorrhage and did not result in a significantly lower risk of cardiovascular disease than placebo (23). Differences between men and women were not analyzed. In a *post hoc* analysis the risk of incident dementia and cognitive decline was analyzed. There was evidence that sex modified the association with incident dementia (interaction *P* = 0.02), with increased risk in men (HR, 1.68; 95% CI, 1.19–2.39) but not women (HR, 1.01; 95% CI, 0.72–1.42) (26).

**The ARRIVE (Use of Aspirin to Reduce Risk of Initial Vascular Events in patients at moderate risk of cardiovascular disease) trial** was a randomized, double-blind, placebo-controlled, multicentre study conducted in seven countries. Eligible patients were aged 55 years (men) or 60 years (women) and older and had an average cardiovascular risk, deemed to be moderate based on the number of specific risk factors. Patients at high risk of gastrointestinal bleeding or other bleeding, or diabetes were excluded. The total number of patients was 12,546 patients (female 29.5%) and they were randomly assigned to receive enteric-coated aspirin tablets (100 mg) or placebo, once daily. Median follow-up was 60 months. The primary efficacy endpoint was a composite outcome of time to first occurrence of cardiovascular death, myocardial infarction, unstable angina, stroke, or transient ischemic attack. In the intention-to-treat analysis, the primary endpoint occurred in 269 (4.29%) patients in the aspirin group vs. 281 (4.48%) patients in the placebo group (HR 0.96; 95% CI 0.81–1.13; *p* = 0.6038). There were 321 documented deaths (*n* = 160 [2.55%] of 6,270 patients in the aspirin group and n=161 [2.57%] of 6,276 patients in the placebo group; HR 0.99, 95% CI 0·80–1.24; *p* = 0.9459). Of these deaths, 108 patients had fatal myocardial infarction, fatal stroke, or other vascular death (*n* = 49 [0.78%] in the aspirin group and *n* = 59 [0.94%] in the placebo group) (24). Gastrointestinal bleeding events occurred in <1% of patients in each group and were predominantly mild but were more frequent for those assigned to aspirin (HR, 2.11; 95%CI, 1.36–3.28, *p* = 0.0007).

From 11 trials only one RCT studied the effect of aspirin in primary prevention in women (WHS), and based on their results aspirin is recommended for primary prevention for women after consideration of the 10-year risk of cerebrovascular disease (CVD) and whether this and age outweigh the risk of hemorrhage (13). In other studies, women were represented relatively well-compared to men (44–70%), except the last 2 trials, where they were underrepresented—ASCEND (37.4%) (21) and ARRIVE (29.5%) (24), however, only in 5 trials men and women were compared with no significant difference (14–18).

#### Meta-Analyses

**Figure 2:**
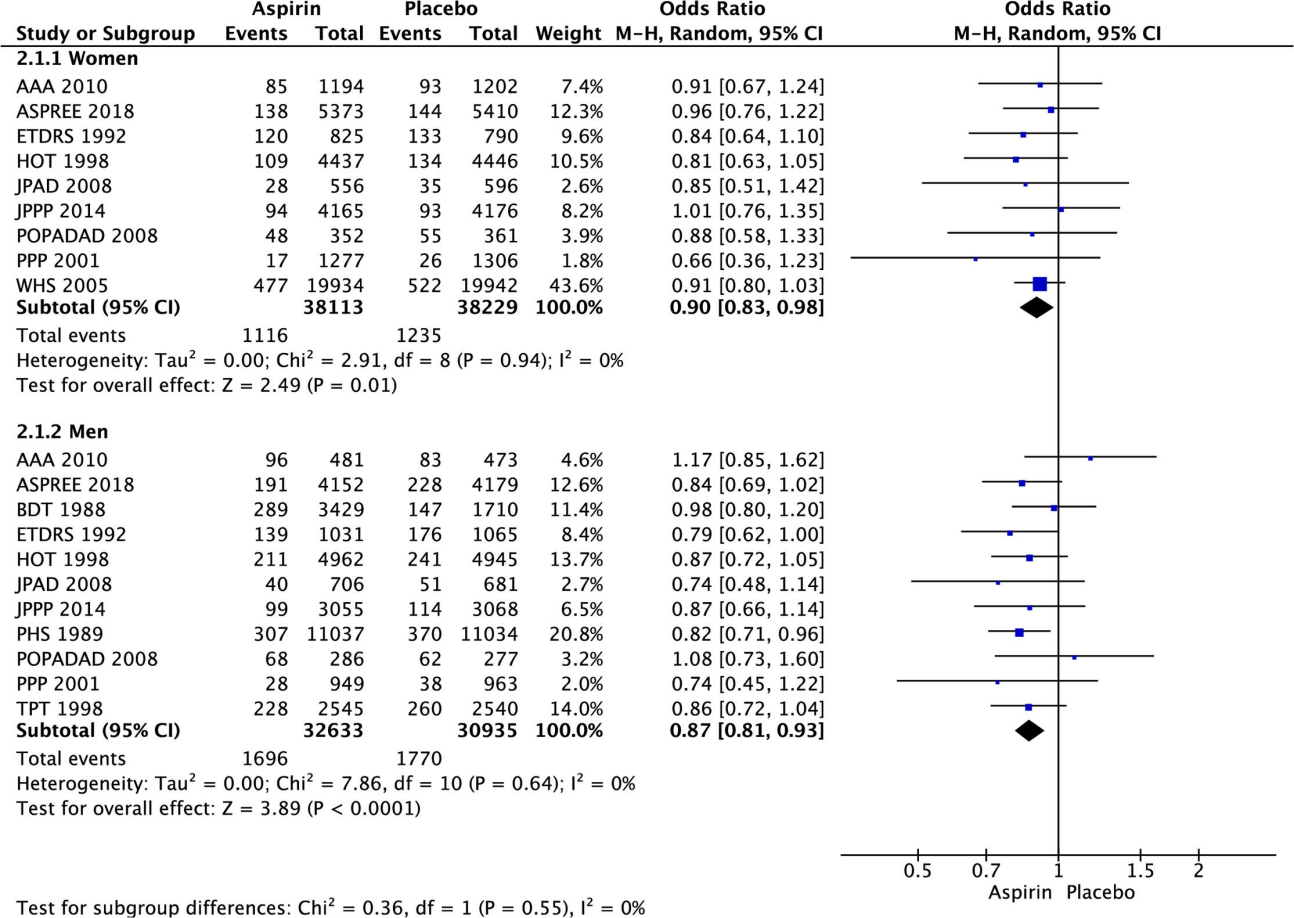
Major cardiovascular events in women and men taking aspirin or placebo.

Meta-analyses of 11 RCTs (135 641 patients) comparing aspirin with placebo showed significant decreases (prevention) in the risk of major cardiovascular events in women and men (Figure 2).

**Figure 3:**
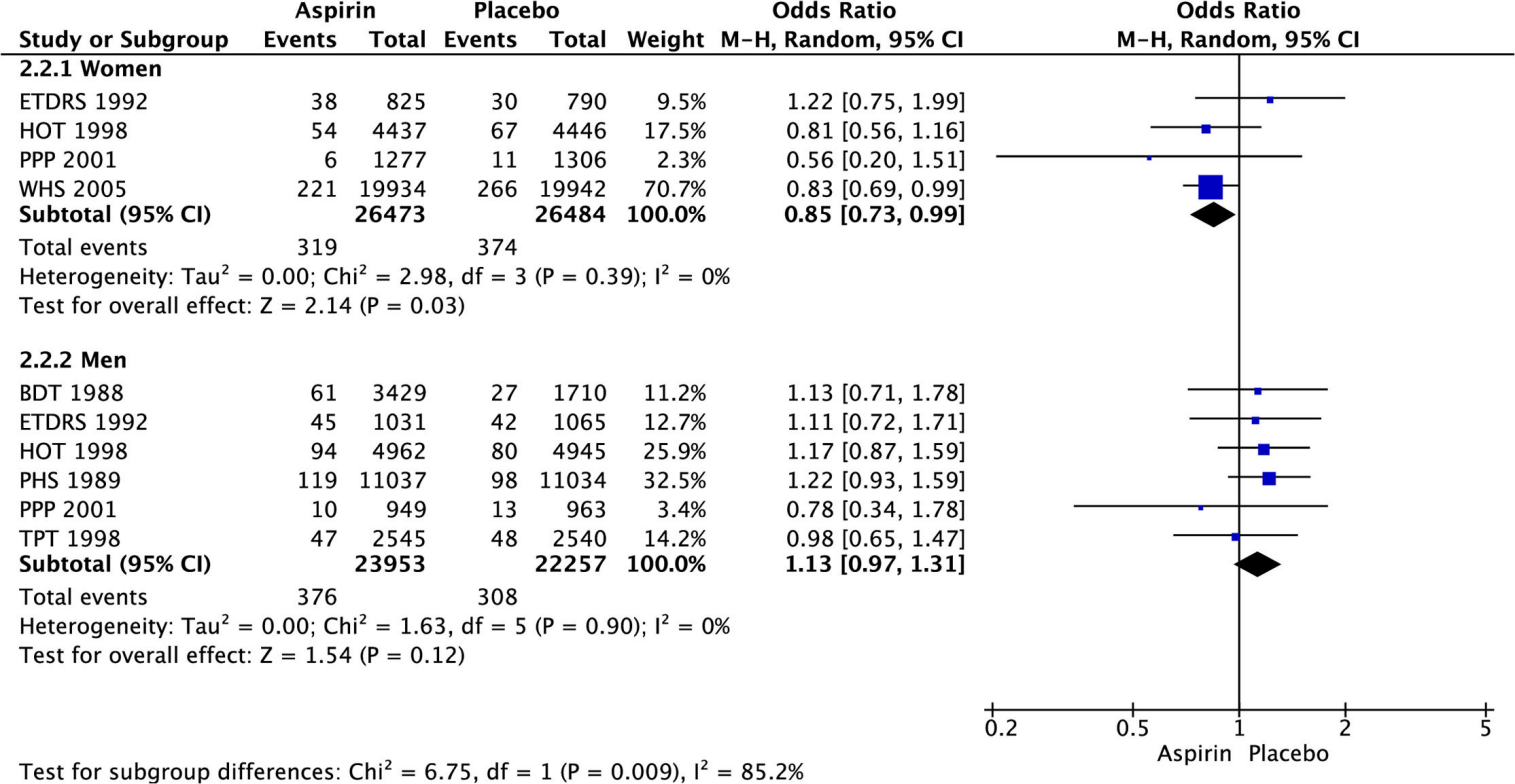
Stroke in women and men taking aspirin or placebo.

**Figure 4:**
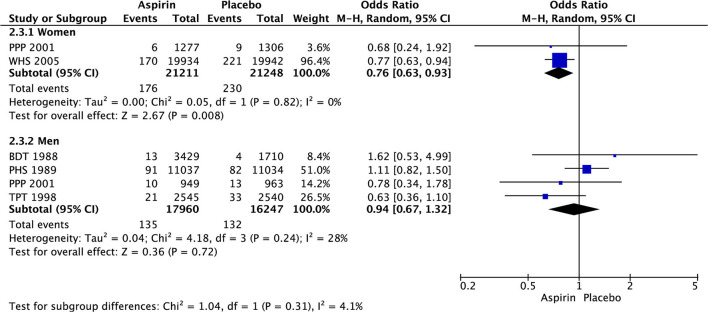
Ischemic stroke in women and men taking aspirin or placebo.

**Figure 5:**
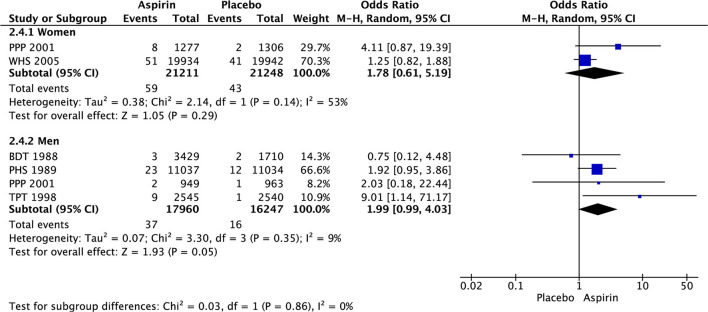
Hemorrhagic stroke in women and men taking aspirin or placebo.

In women, there was significant decrease in the risk of stroke (OR 0.85 [95% CI 0.73, 0.99], *p* = 0.03) (Figure 3) and ischemic stroke (OR 0.76 [0.63, 0.93], *p* = 0.008) (Figure 4) with aspirin compared to placebo and there was no significant increase in risk of hemorrhagic stroke (OR 1.78 [0.61, 5.19], *p* = 0.29) (Figure 5) with aspirin compared to placebo. Aspirin also significantly decreased the risk of transient ischemic attack (OR 0.78 [0.64, 0.95], *p* = 0.01), compared to placebo, though only one study was included in the analysis (Table 2).

**Table 2:** Role of aspirin in primary prevention of stroke, major cardiovascular events, mortality, myocardial infarction (MI), and bleeding in women and men.

**Outcome**	**Incidence (%)**		***n* (*N*)**	**OR [95% CI]**	***I* ^2^**	***P*-value**
	**Aspirin**	**Placebo**				
**Major cardiovascular events**
Women	2.9%	3.2%	9 (76,342)	0.90 [0.83, 0.98]	0%	0.01
Men	5.2%	5.7%	11 (63,568)	0.87 [0.81, 0.93]	0%	<0.0001
**Stroke**
Women^a^	1.2%	1.4%	4 (52,957)	0.85 [0.73, 0.99]	0%	0.03
Men	1.6%	1.4%	6 (46,210)	1.13 [0.97, 1.31]	0%	0.12
**Ischemic stroke**
Women	0.8%	1.1%	2 (42,459)	0.76 [0.63, 0.93]	0%	0.008
Men	0.8%	0.8%	4 (34,207)	0.94 [0.67, 1.32]	28%	0.72
**Hemorrhagic stroke**
Women	0.3%	0.2%	2 (42,459)	1.78 [0.61, 5.19]	53%	0.29
Men	0.2%	0.1%	4 (34,207)	1.99 [0.99, 4.03]	9%	0.05
**TIA**
Women	0.9%	1.2%	1 (39,876)	0.78 [0.64, 0.95]	NA	0.01
Men	NR	NR	NR	NR	NR	NR
**Mortality**
Women	3.4%	3.5%	5 (63,740)	0.92 [0.77, 1.10]	66%	0.37
Men	4.8%	4.7%	7 (54,541)	0.97 [0.87, 1.08]	39%	0.57
**Cardiovascular mortality**
Women	1.0%	1.1%	5 (53,670)	0.90 [0.74, 1.09]	15%	0.26
Men	2.2%	2.1%	7 (46,773)	0.97 [0.85, 1.10]	4%	0.64
**Myocardial infarction**
Women	1.2%	1.3%	4 (52,957)	0.92 [0.77, 1.11]	13%	0.38
Men^b^	2.2%	3.2%	6 (46,210)	0.68 [0.58, 0.81]	47%	<0.0001
**Vascular events/ revascularization**
Women	9.0%	9.6%	1 (5,796)	0.93 [0.78, 1.11]	NA	0.42
Men	11.8%	13.6%	1 (9,684)	0.85 [0.76, 0.96]	NA	0.009
**Coronary revascularisation**
Women	2.0%	1.9%	1 (39,876)	1.04 [0.90, 1.20]	NA	0.58
Men	NR	s	NR	NR	NR	NR
**Major bleeding**
Women	3.7%	2.8%	5 (67,921)	1.43 [1.19, 1.72]	52%	0.0002
Men	3.3%	2.5%	8 (84,200)	1.37 [1.26, 1.49]	0%	<0.00001
**GIT bleeding**
Women	0.6%	0.5%	1 (39,876)	1.40 [1.07, 1.83]	NA	0.01
Men	3.4%	3.2%	2 (27,156)	1.05 [0.92, 1.20]	0%	0.46
**Cardiovascular outcome**
Women	3.9%	3.8%	1 (10783)	1.05 [0.86, 1.28]	NA	0.64
Men	5.7%	6.5%	1 (8331)	0.87 [0.73, 1.04]	NA	0.14

*CI, Confidence interval; GIT, Gastrointestinal; I^2^, Heterogeneity; MI, myocardial infarction, n, Number of studies; N, Number of patients; NA, Not applicable; NR, Not reported; p, Statistical significance value; OR, Odds Ratio; TIA, transient ischemic attack,*

a *: Women vs. Men (improvement of outcome with aspirin; P = 0.009);*

b *: Men vs. Women (improvement of outcome with aspirin; p = 0.02)*.

In men, aspirin did not affect the risk of stroke (OR 1.13 [0.97, 1.31], *p* = 0.12) (Figure 3) and ischemic stroke (OR 0.94 [0.67, 1.32], *p* = 0.72) (Figure 4), but increased the risk of hemorrhagic stroke with a borderline statistical significance (OR 1.99 [0.99, 4.03], *p* = 0.05) (Figure 5) compared to placebo (Table 2). There were no marked effects of aspirin compared to placebo on overall mortality and cardiovascular mortality in women and men.

**Figure 6:**
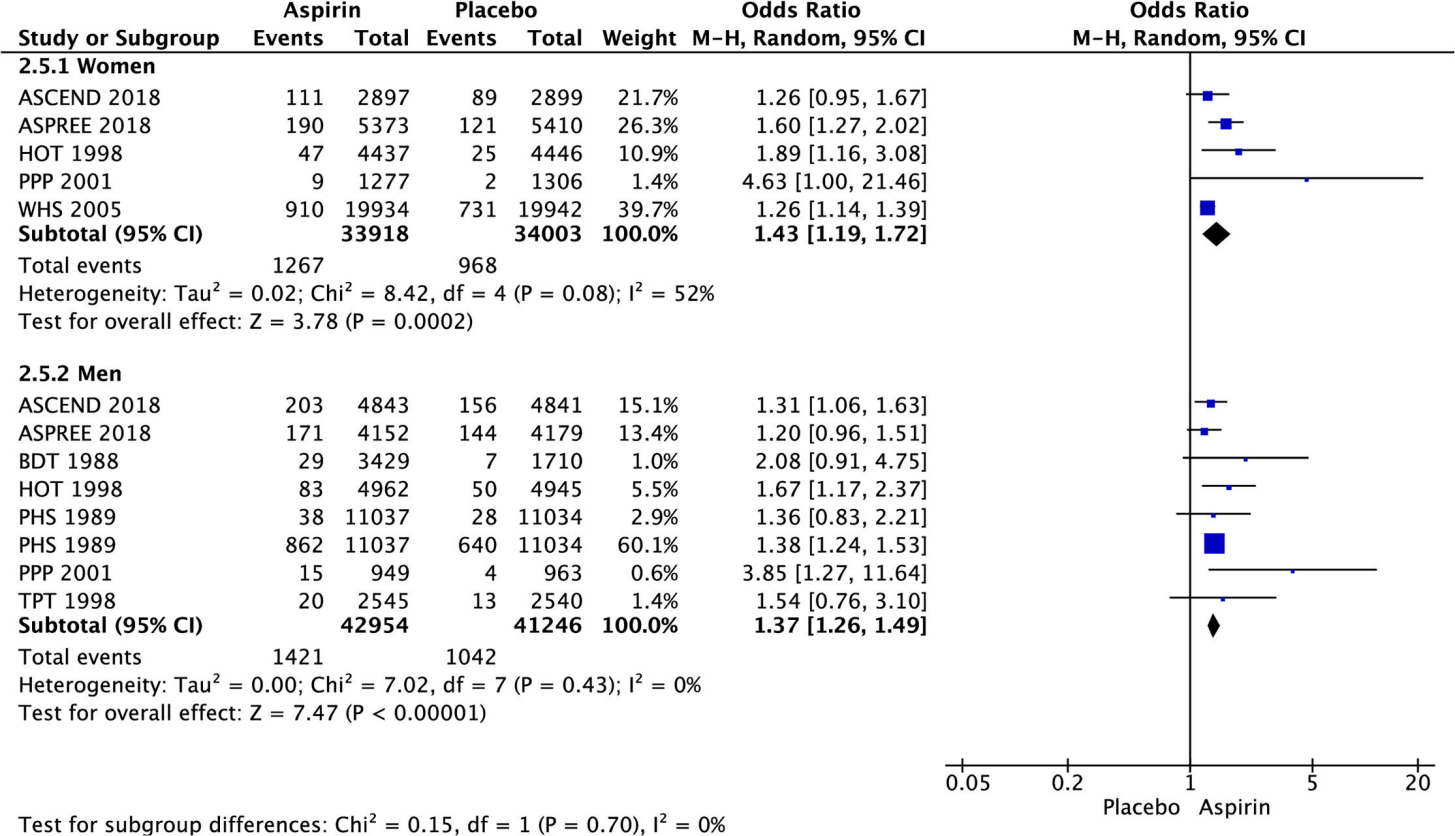
Major bleeding in women and men taking aspirin or placebo.

**Figure 7:**
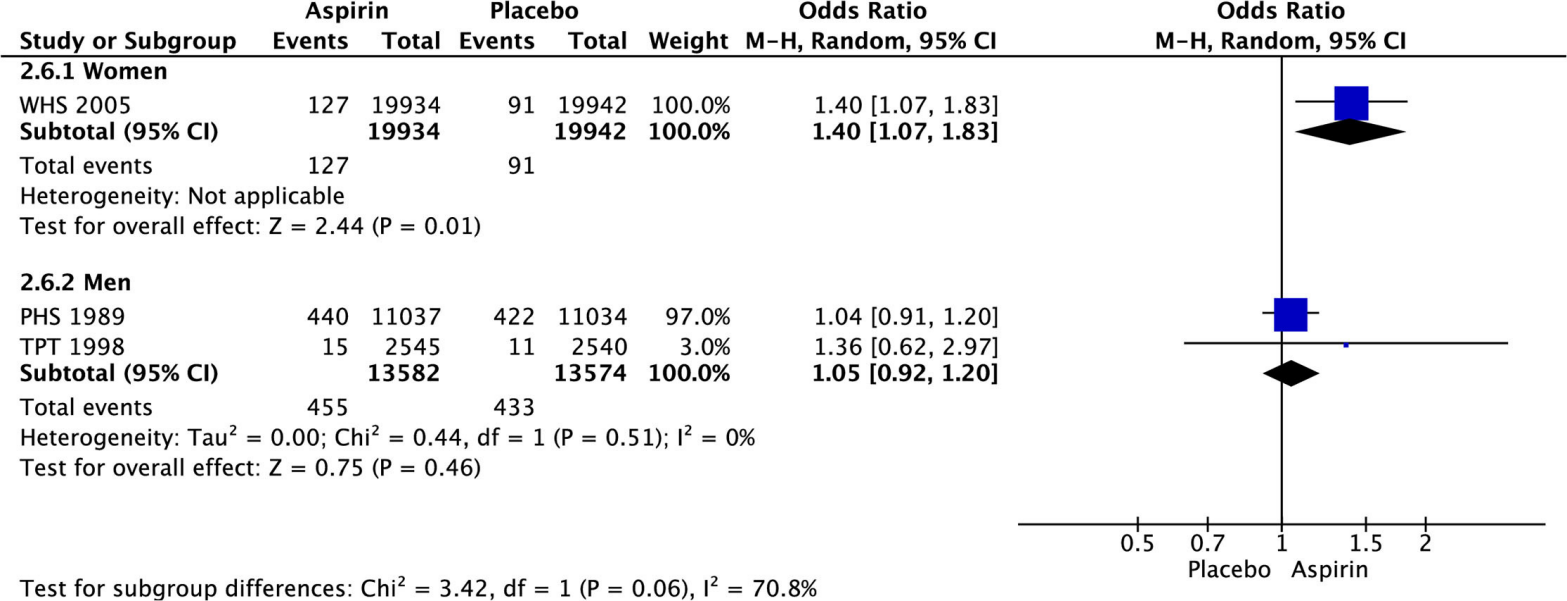
Gastrointestinal bleeding in women and men taking aspirin or placebo.

Compared to placebo, aspirin significantly increased the major bleeding in both, women (OR 1.43 [1.19, 1.72], *p* = 0.002) and men (OR1.37 [1.26, 1.49], *p* < 0.00001) (Figure 6), while gastrointestinal (GIT) bleeding was significantly increased only in women (OR 1.40 [1.07, 1.83], *p* = 0.01) (Figure 7) taking aspirin (Table 2).

In women, aspirin did not affect the risk of myocardial infarction (OR 0.92 [0.77, 1.11], *p* = 0.38) while in men aspirin significantly decreased this risk (OR 0.68 [0.58, 0.81], *p* < 0.0001) compared to placebo (Table 2).

Mortality was not reduced in either women or men taking aspirin (Table 2).

**Figure 8:**
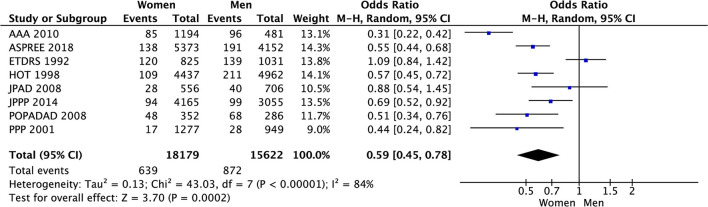
Major cardiovascular events in women and men taking aspirin.

**Figure 9:**
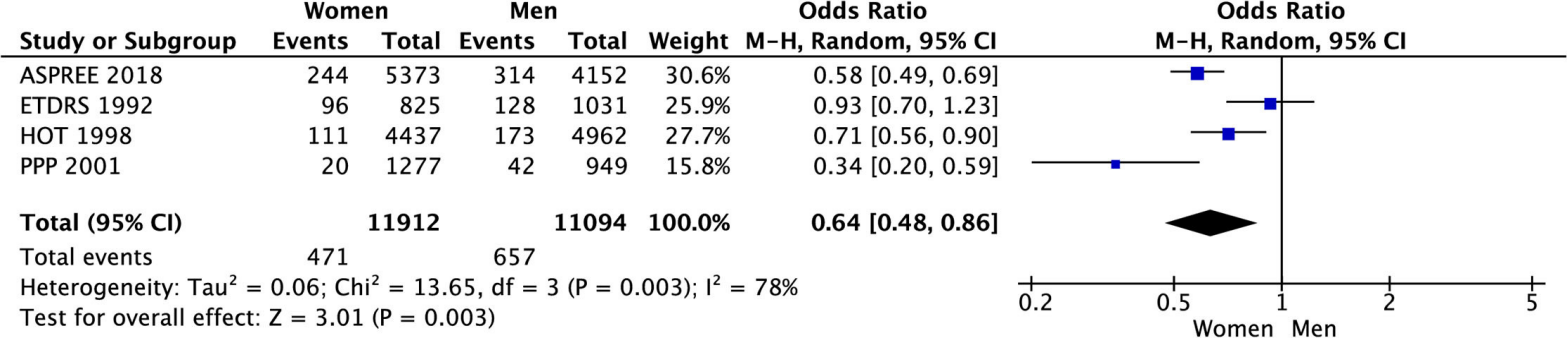
Mortality in women and men taking aspirin.

**Figure 10:**
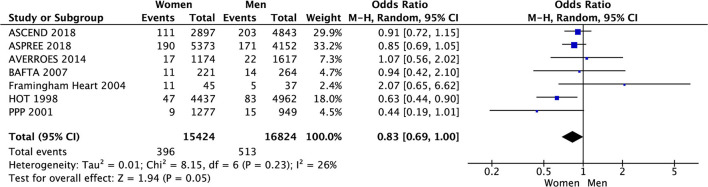
Major bleeding in women and men taking aspirin.

**Figure 11:**
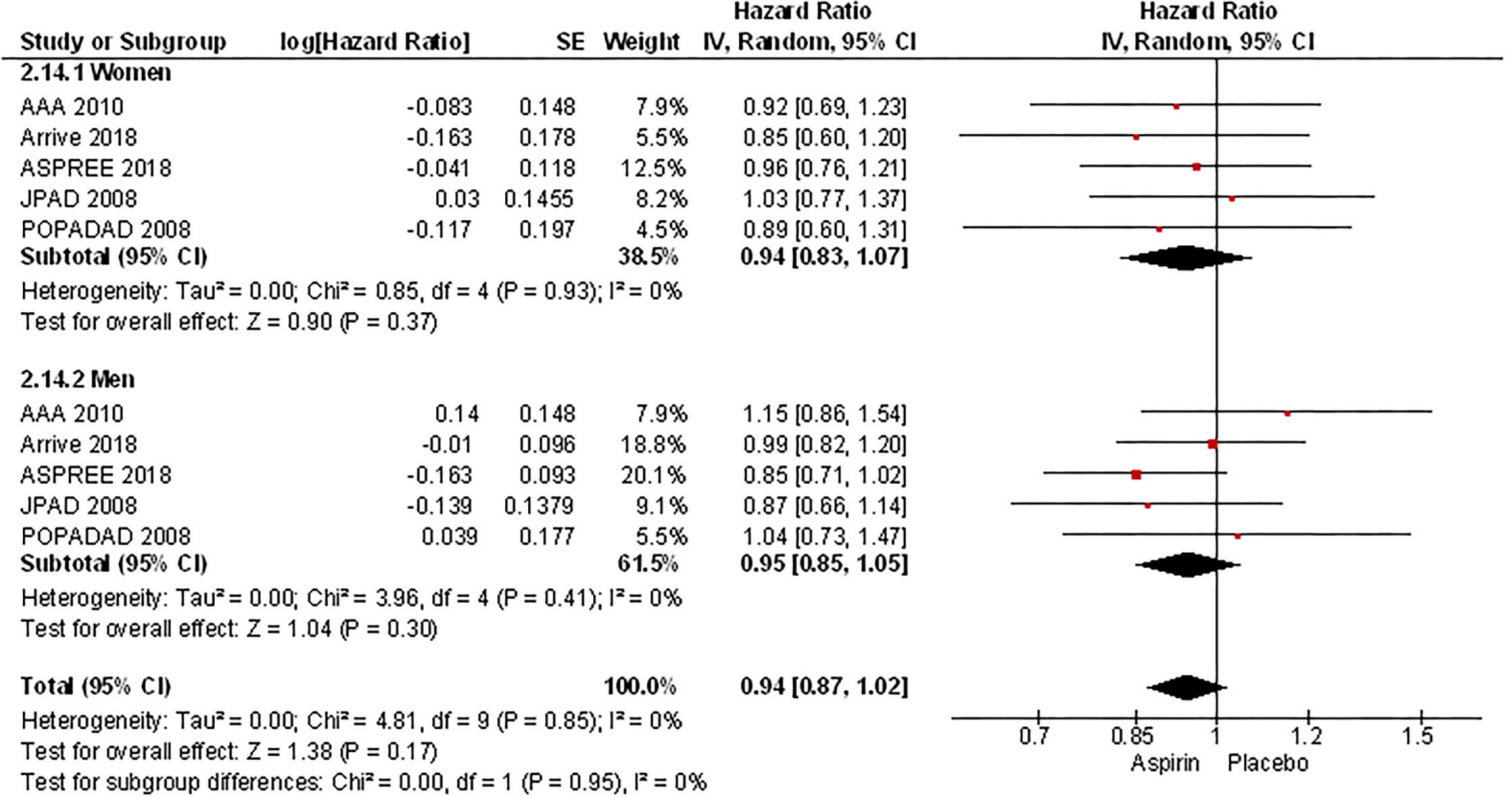
Hazard ratio of composite outcome women and men taking aspirin.

**Figure 12:**
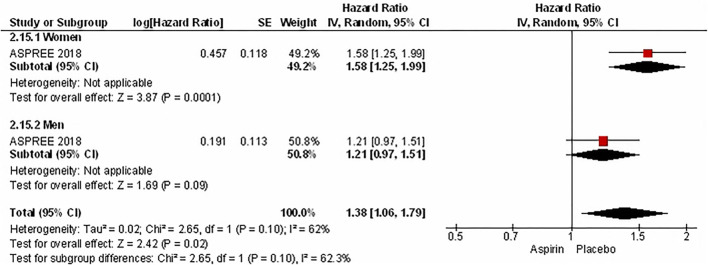
Hazard ratio of major bleeding in women and men taking aspirin.

In the meta-analysis of event rates in women and men taking aspirin, the risk of major cardiovascular events (3.5% vs. 5.6%, OR 0.59 [0.45, 0.78], *p* = 0.0002) (33 801 patients) in 8 RCTs (Figure 8), mortality (4.0% vs. 5.9%, OR 0.64 [0.48, 0.86], *p* = 0.003) in 23 006 patients from 4 RCTs (Figure 9), and major bleeding (2.6% vs. 3.0%, OR 0.83 [0.69, 1.00], *p* = 0.05) in 32 248 patients from 7 RCTs (Figure 10) (Table 3) were significantly lower in women compared to men (Table 3). On meta-analyzing the Hazard ratio, there was no marked effect of aspirin on composite outcome compared to placebo in women HR 0.94 [0.83, 1.07] and men HR 0.95 [0.85, 1.05]. In addition, there was no difference in the effect of aspirin vs. placebo in women and men, *p* = 0.95. There was no statistically significant heterogeneity among the studies (Figure 11). The HR of major bleeding was significantly greater in women taking aspirin, HR 1.58 [1.25, 1.99], *p* = 0.0001 and tend to be greater in men taking aspirin, HR 1.21 [0.97, 1.51], though data was not statistically significant. Compared to placebo, there was no significant difference in the effect of aspirin in women and men on the risk of major bleeding (*p* = 0.10) (Figure 12).

**Table 3:** Role of aspirin in primary prevention of MACE, stroke, mortality, and bleeding in Women compared with Men.

**Outcome**	**Incidence (%)**		***n* (N)**	**OR [95% CI]**	***I* ^2^**	***P*-value**
	**Women**	**Men**				
Major cardiovascular events	3.5%	5.6%	8 (33,801)	0.59 [0.45, 0.78]	84%	0.0002
Stroke	1.5%	2.1%	3 (13,481)	0.74 [0.48, 1.14]	53%	0.17
Ischemic stroke	2.4%	2.0%	2 (5, 017)	0.96 [0.25, 3.73]	84%	0.96
Hemorrhagic stroke	0.6%	0.2%	1 (2,226)	2.99 [0.63, 14.09]	NA	0.17
Mortality	4.0%	5.9%	4 (23, 006)	0.64 [0.48, 0.86]	78%	0.003
Cardiovascular mortality	2.3%	2.9%	4 (14,119)	0.71 [0.44, 1.12]	72%	0.14
Myocardial infarction	1.8%	2.2%	3 (13,481)	0.77 [0.45, 1.32]	70%	0.35
Major bleeding	2.6%	3.0%	7 (32,248)	0.83 [0.69, 1.00]	26%	0.05
Major and clinically relevant bleeding	4.9%	4.5%	2(2,873)	1.19 [0.58, 2.44]	47%	0.64
Intracranial bleeding	0.6%	0.4%	2(2,873)	1.28 [0.45, 3.60]	0%	0.64
Stroke and systemic embolism	5.5%	3.0%	1(2,791)	1.85 [1.26, 2.70]	NA	0.002
Primary outcome	9.5%	10.1%	1(488)	0.93 [0.51, 1.70]	NA	0.82
Cardiovascular disease	3.9%	5.7%	1 (9,525)	0.68 [0.56, 0.82]	NA	<0.0001
Vascular event/Revascularization	9.0%	11.8%	1 (7,740)	0.73 [0.63, 0.86]	NA	<0.0001

*CI, Confidence interval; I^2^, Heterogeneity; n, Number of studies; N, Number of patients; NA, Not applicable; p, Statistical significance value; OR, Odds Ratio; Primary outcome: Fatal or non-fatal disabling stroke (ischemic or hemorrhagic), intracranial haemorrhage, or clinically significant arterial embolism*.

There were no differences in event rates between women and men taking aspirin for stroke, ischemic stroke or hemorrhagic stroke, cardiovascular mortality, or myocardial infarction. For hemorrhagic stroke, stroke and systemic embolism, cardiovascular disease and vascular event/revascularisation there was only one RCT for each outcome, so we cannot draw and generalize any conclusions from these results; moreover, no differences were found between men and women in terms of risk of hemorrhagic stroke and primary outcome in these RCTs (Table 3).

**Figure 13:**
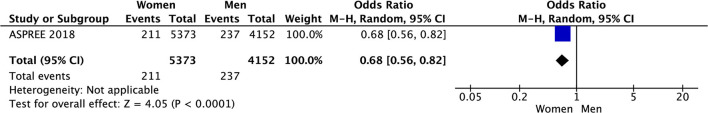
Cardiovascular disease in women and men taking aspirin.

**Figure 14:**
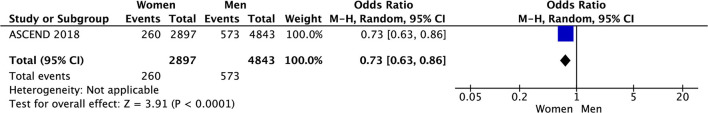
Vascular event/Revascularization in women and men taking aspirin.

In ASPREE the risk of cardiovascular disease was significantly decreased in women compared to men (3.9% vs. 5.7%, OR 0.68 [0.56, 0.82], *p* < 0.0001) in 9 525 patients (Figure 13) and in the ASCEND trial the risk of vascular event/revascularization was also significantly decreased in women (9.0% vs. 11.8%, OR 0.73 [0.63, 0.86], *p* < 0.0001) in 7 740 of patients (Figure 14), however, representation of women in ASCEND was only 37.4%.

### Study of Aspirin on Risk of Ischemic Stroke in Patients With Covert Cerebral Small Vessel Disease (CcSVD)

Another study sought to detect if aspirin reduces the risk of ischemic stroke or increases hemorrhagic stroke risk in patients with covert cerebral small vessel disease (ccSVD). Only one RCT included patients aged ≥ 45 years with at least one silent brain infarct (SBI) but no previous clinical cerebrovascular events for randomization to aspirin 100 mg (36 patients) or placebo (47 patients). The primary endpoint was the combined endpoint of ischemic stroke, TIA, and new silent brain infarcts detected by MRI which had occurred in 9 controls (19.1%) and in two subjects (5.6%) in the ASA group (*p* = 0.10) after 4 years (26). A new stroke was observed in 1/36 (2.8%) in the ASA group compared to 2/47 (4.3%) subjects in control group (OR 0.64 [0.06–7.38]) There were no (0/36 [0%]) deaths in the aspirin-treated group and 1/47 (2.1%) death in the control group (O: 0.64 [0.06–7.38]) (11, 25), gastrointestinal adverse effects were reported in 2 (5.6%) aspirin-treated patients compared to 1 (2.1%) in the control group. There was no significant difference in the incidence of cognitive impairment between treated and non-treated patients during the 4-year follow-up (25). The findings should be interpreted with caution, taking into account a small sample of the population investigated, the imbalance in the prevalence of hypertension between the aspirin-treated patient group (17/36 [47.2%]) and the control group (29/47 [61.7%]) (*p* = 0.188) and, in relation to cognitive impairment, also the relatively young age (median of 66 years in the aspirin group vs. 68 years in the control group) and the relatively short period of follow-up.

## Discussion

In primary prevention, the role of aspirin remains controversial and net benefit less certain for women, moreover only in five studies women and men were compared (14–18).

In a meta-analysis of 11 trials enrolling 135 641 participants in primary prevention with aspirin we found a significant decrease in the risk of major cardiovascular events in both sexes with a decreased risk of stroke and ischemic stroke in women taking aspirin compared to placebo with no significantly increased risk of hemorrhagic stroke.

In men, aspirin did not affect the risk of stroke and ischemic stroke, but significantly increased the risk of hemorrhagic stroke compared to placebo.

Compared to placebo, aspirin significantly increased major bleeding in both, men and women, while gastrointestinal (GIT) bleeding was significantly increased only in women receiving aspirin.

In women, aspirin did not affect the risk of MI while in men it significantly decreased the risk of MI compared to placebo. These findings are consistent with other published meta-analyses on the effect of aspirin on primary stroke prevention (27–29). In meta-analysis of 13 trials, Zheng et al. found that the use of aspirin in individuals without cardiovascular disease was associated with a lower risk of cardiovascular events and with an increased risk of major bleeding, and that the number needed to treat to cause major bleeding was lower than number needed to treat to prevent an ASCVD (atherosclerotic cardiovascular disease) event (210 vs. 265) (27). Based on this, according to the authors, the use of aspirin indicates more harm than benefit (27). This was confirmed by a meta-analysis published by Lei et al. (28) showing that in healthy adults and patients with cardiovascular diseases the little protective benefit from aspirin is offset by the increased risk of severe bleeding events (28).

Therefore, even the current guidelines recommend aspirin for, primary prevention only with restrictions and the strength of the recommendations is mostly weak (30).

Recently published trials (ASCEND, ASPREE, ARRIVE) were focused on the effect of aspirin in primary prevention in three risk groups of patients, i.e., patients with diabetes mellitus, older patients without life-limiting chronic illness, and patients at intermediate risk of a future atherosclerotic event (21, 22, 24). The meta-analysis of the former three trials did not report a significant survival benefits with aspirin in primary prevention (risk ratio, 0.98; 95% CI, 0.93–1.02, *p* = 0.30) and confirmed the increased risk of major bleeding (risk ratio, 1.47; 95% CI, 1.31–1.65; *p* < 0.0001 (29).

The effect of aspirin in primary prevention in women was specifically addressed by only one RCT (WHS) and similarly only 1 meta-analysis (from 2006) was focused on determining if the benefits and risks of the treatment with aspirin in primary prevention of cardiovascular disease vary by sex (31). Based on WHS trial, the most consistent benefit for aspirin was in women ≥ 65 years of age at study entry (14). According to a meta-analysis, for both women and men, aspirin therapy reduced the risk of a composite of cardiovascular events due to its effect on reducing the risk of ischemic stroke in women and MI in men and significantly increased the risk of bleeding to a similar degree among women and men. When the benefits and risks for women were compared, the average absolute benefit in women receiving aspirin therapy was ~2 stroke events per 1,000 women treated and the risk of major bleeding was 2.5 per 1,000 treated women (31). The Southern Community Cohort Study analyzed low-dose aspirin in primary prevention by race/ethnicity and found decreased risk of ischemic cardiac death in white participants, especially women. The history of peptic ulcer was not associated with low-dose aspirin, whereas it was associated to the concomitant use of NSAIDs (32).

According the American Heart Association Effectiveness-Based Guidelines for Prevention of cardiovascular Disease in Women-2011 Update: (i) aspirin (75–325 mg) is deemed reasonable to be used in women with diabetes mellitus without known CVD unless contraindicated; (ii) aspirin therapy can be useful in women ≥ 65 years of age without known CVD (81 mg/d or 100 mg every other day) if blood pressure is controlled and the benefit for ischemic stroke and myocardial infarction prevention is likely to outweigh risk of gastrointestinal bleeding and hemorrhagic stroke; iii) aspirin (81 mg/d or 100 mg every other day) may be reasonable for ischemic stroke prevention in women aged < 65 years without known CVD (1).

In our meta-analysis on the role of aspirin in primary prevention in men and women, we found that the risk of major cardiovascular events, mortality and major bleeding were significantly lower in women compared to men.

There were no marked differences between women and men taking aspirin regarding the risk of stroke, ischemic stroke or hemorrhagic stroke, cardiovascular mortality, or myocardial infarction. However, for hemorrhagic stroke, stroke and systemic embolism, primary outcome, cardiovascular disease and vascular event/revascularisation there was only one RCT for each outcome.

In the ASPREE trial, in patients aged > 75 years the risk of cardiovascular disease was significantly decreased in women compared to men. This confirmed the results from the WHS trial, where the most consistent benefit for aspirin was in women ≥ 65 years of age at study entry, among whom the risk of major cardiovascular events was reduced by 26% (20). In the ASCEND trial the risk of vascular event/revascularisation was also significantly decreased in women but representation of women in ASCEND was only 37.4% (21).

Although we have found significant decreases in the primary prevention of the risk of major cardiovascular events in both women and men and in women a decreased risk of stroke and ischemic stroke with aspirin compared to placebo with no significantly increased risk of hemorrhagic stroke, it is apparent that this benefit is associated with an overall increased risk of bleeding. Indeed, aspirin significantly increased the major bleeding in both men and women. In contrast, gastrointestinal (GIT) bleeding was significantly increased only in women treated with aspirin.

Some works reported that co-prescription of aspirin with a proton pump inhibitor (PPI) might limit the risk of significant gastrointestinal bleeding; the use of PPIs was inconsistently reported in most of the studies and this strategy has not been adequately tested in RCTs (27). Therefore, we do not have sufficient evidence to recommend a concomitant treatment with PPI to reduce the risk of bleeding.

With regards to patient care, it should be considered that most of the cited trials were conducted in an era where pharmacological preventive measures available nowadays were less widespread (e.g., lipid lowering therapies). Therefore, the results of these aspirin trials, which showed minimal benefits and consistent bleeding risks, should be considered alongside the results of statin trials (33), recommendations for blood pressure control (34) and treatment of diabetes mellitus (35). In primary prevention trials, the use of statins was associated with a decreased risk of major vascular events (36). This statistically and clinically relevant benefit was associated with a favorable safety profile, in particular, it was not associated with the bleeding complications observed with aspirin (33). Given the increased cardiovascular risk, all patients with DM require aggressive risk factor reduction. In primary prevention, a combination of tight controls of blood pressure, lipids and diabetes is certainly crucial. However, studies have consistently shown that women are underdiagnosed and undertreated compared with men (37, 38). Furthermore, women with DM have poorer blood pressure, lipid and DM control compared with their male counterparts (35, 39).

We found no evidence to support the use of aspirin in patients with ccSVD which is in line with the ESO guideline on covert cerebral small vessel disease published in 2021 (25), where, given the low quality of evidence, only Expert Consensus Statement was formulated as follows: (i) Most group members advised against the use of antiplatelet drugs to prevent clinical outcomes in subjects with ccSVD when no other indication for this treatment exists; (ii) with current available knowledge, the use of antiplatelet drugs to prevent progression of cerebral SVD may be harmful in older patients (from around ≥ 70 years of age) if no other indication for this treatment exists (11).

## Conclusion

In both women and men aspirin reduced the risk of major cardiovascular events and in women it decreased the risk of stroke and ischemic stroke with no significantly increased risk of hemorrhagic stroke.

In men, aspirin did not affect the risk of stroke and ischemic stroke but significantly increased the risk of hemorrhagic stroke compared to placebo. Aspirin increased the risk of major bleeding in both women and men, while gastrointestinal bleeding was significantly increased only in women treated with aspirin.

Based on the current ESO guidelines, aspirin is not recommended in covert cerebral small vessel disease.

## Author Contributions

All authors listed have made a substantial, direct, and intellectual contribution to the work and approved it for publication.

## Conflict of Interest

The authors declare that the research was conducted in the absence of any commercial or financial relationships that could be construed as a potential conflict of interest.

## Publisher's Note

All claims expressed in this article are solely those of the authors and do not necessarily represent those of their affiliated organizations, or those of the publisher, the editors and the reviewers. Any product that may be evaluated in this article, or claim that may be made by its manufacturer, is not guaranteed or endorsed by the publisher.

## References

[1] Mosca L Benjamin EJ Berra K Bezanson JL Dolor RJ Lloyd-Jones DM. Effectiveness-based guidelines for the prevention of cardiovascular disease in women−2011 update: a guideline from the American Heart Association. J Am Coll Cardiol. (2011) 57:1404–23. 10.1016/j.jacc.2011.02.005 21388771 PMC3124072

[2] Bell AD Roussin A Cartier R Chan WS Douketis JD Gupta A. The use of antiplatelet therapy in the outpatient setting: Canadian cardiovascular society guidelines executive summary. Can J Cardiol. (2011) 27:208–21. 10.1016/j.cjca.2010.12.033 21459270

[3] Piepoli MF Hoes AW Agewall S Albus C Brotons C Catapano AL. 2016 European Guidelines on cardiovascular disease prevention in clinical practice: the Sixth Joint Task Force of the European Society of Cardiology and Other Societies on Cardiovascular Disease Prevention in Clinical Practice (constituted by representatives of 10 societies and by invited experts) Developed with the special contribution of the European Association for Cardiovascular Prevention & Rehabilitation (EACPR). Eur Heart J. (2016) 37:2315–81. 10.1093/eurheartj/ehw106 27222591 PMC4986030

[4] Bibbins-Domingo K Force USPST. Aspirin use for the primary prevention of cardiovascular disease and colorectal cancer: U.S. Preventive Services Task Force recommendation statement. Ann Intern Med. (2016) 164:836–45. 10.7326/M16-0577 27064677

[5] Mehta SR Bainey KR Cantor WJ Lordkipanidzé M Marquis-Gravel G Robinson SD. 2018 Canadian Cardiovascular Society/Canadian Association of Interventional Cardiology focused update of the guidelines for the use of antiplatelet therapy. Can J Cardiol. (2018). 34:214–33. 10.1016/j.cjca.2017.12.012 29475527

[6] Arnett DK Khera A Blumenthal RS. 2019 ACC/AHA Guideline on the primary prevention of cardiovascular disease: part 1, lifestyle and behavioral factors. JAMA Cardiol. (2019) 4:1043–4. 10.1001/jamacardio.2019.2604 31365022

[7] American Diabetes Association Professional Practice Committee. 10. Cardiovascular disease and risk management. Diabetes Care. (2019) 42(Suppl. 1):S103–23. 10.2337/dc19-S010 30559236

[8] Ajayi AA Mathur R Halushka PV. Testosterone increases human platelet thromboxane A2 receptor density and aggregation responses. Circulation. (1995) 91:2742–7. 10.1161/01.CIR.91.11.2742 7758179

[9] Feuring M Christ M Roell A Schueller P Losel R Dempfle CE. Alterations in platelet function during the ovarian cycle. Blood Coagul Fibrinolysis. (2002) 13:443–7. 10.1097/00001721-200207000-00009 12138372

[10] Caso V Santalucia P Acciarresi M Pezzella FR Paciaroni M. Antiplatelet treatment in primary and secondary stroke prevention in women. Eur J Intern Med. (2012) 23:580–5. 10.1016/j.ejim.2012.04.010 22939800

[11] Wardlaw JM Debette S Jokinen H De Leeuw FE Pantoni L Chabriat H. ESO Guideline on covert cerebral small vessel disease. Eur Stroke J. (2021) 6:IV. 10.1177/23969873211027002 34414305 PMC8370062

[12] Page MJ McKenzie JE Bossuyt PM Boutron I Hoffmann TC Mulrow CD. The PRISMA 2020 statement: an updated guideline for reporting systematic reviews. Rev Esp Cardiol. (2021) 74:790–9. 10.1016/j.rec.2021.07.010 34446261

[13] Ridker PM Cook NR Lee IM Gordon D Gaziano JM Manson JE. A randomized trial of low-dose aspirin in the primary prevention of cardiovascular disease in women. N Engl J Med. (2005) 352:1293–304. 10.1056/NEJMoa050613 15753114

[14] Fowkes FG Price JF Stewart MC Butcher I Leng GC Pell AC. Aspirin for prevention of cardiovascular events in a general population screened for a low ankle brachial index: a randomized controlled trial. JAMA. (2010) 303:841–8. 10.1001/jama.2010.221 20197530

[15] ETDRSDaBPCERN. Early treatment diabetic retinopathy study design and baseline patient characteristics: ETDRS report number 7. Ophthalmology. (1991) 98:741–56. 10.1016/S0161-6420(13)38009-9 2062510

[16] Ogawa H Nakayama M Morimoto T Uemura S Kanauchi M Doi N. Low-dose aspirin for primary prevention of atherosclerotic events in patients with type 2 diabetes: a randomized controlled trial. JAMA. (2008) 300:2134–41. 10.1001/jama.2008.623 18997198

[17] Belch J MacCuish A Campbell I Cobbe S Taylor R Prescott R. The prevention of progression of arterial disease and diabetes (POPADAD) trial: factorial randomised placebo controlled trial of aspirin and antioxidants in patients with diabetes and asymptomatic peripheral arterial disease. BMJ. (2008) 337:a1840. 10.1136/bmj.a1840 18927173 PMC2658865

[18] Ikeda Y Shimada K Teramoto T Uchiyama S Yamazaki T Oikawa S. Low-dose aspirin for primary prevention of cardiovascular events in Japanese patients 60 years or older with atherosclerotic risk factors: a randomized clinical trial. JAMA. (2014) 312:2510–20. 10.1001/jama.2014.15690 25401325

[19] Hansson L Zanchetti A Carruthers SG Dahlöf B Elmfeldt D Julius S. Effects of intensive blood-pressure lowering and low-dose aspirin in patients with hypertension: principal results of the Hypertension Optimal Treatment (HOT) randomised trial. HOT Study Group. Lancet. (1998) 351:1755–62. 10.1016/S0140-6736(98)04311-6 9635947

[20] Sacco M Pellegrini F Roncaglioni MC Avanzini F Tognoni G Nicolucci A. Primary prevention of cardiovascular events with low-dose aspirin and vitamin E in type 2 diabetic patients: results of the Primary Prevention Project (PPP) trial. Diabetes Care. (2003) 26:3264–72. 10.2337/diacare.26.12.3264 14633812

[21] Bowman L Mafham M Stevens W Haynes R Aung T Chen F. ASCEND: a study of cardiovascular events in diabetes: characteristics of a randomized trial of aspirin and of omega-3 fatty acid supplementation in 15,480 people with diabetes. Am Heart J. (2018) 198:135–44. 10.1016/j.ahj.2017.12.006 29653635 PMC5971211

[22] McNeil JJ Woods RL Nelson MR Reid CM Kirpach B Wolfe R. Effect of aspirin on disability-free survival in the healthy elderly. N Engl J Med. (2018) 379:1499–508. 10.1056/NEJMoa1800722 30221596 PMC6426126

[23] McNeil JJ Wolfe R Woods RL Tonkin AM Donnan GA Nelson MR. Effect of aspirin on cardiovascular events and bleeding in the healthy elderly. N Engl J Med. (2018) 379:1509–18. 10.1056/NEJMoa1805819 30221597 PMC6289056

[24] Gaziano JM Brotons C Coppolecchia R Cricelli C Darius H Gorelick PB. Use of aspirin to reduce risk of initial vascular events in patients at moderate risk of cardiovascular disease (ARRIVE): a randomised, double-blind, placebo-controlled trial. Lancet. (2018) 392:1036–46. 10.1016/S0140-6736(18)31924-X 30158069 PMC7255888

[25] Maestrini I Altieri M Di Clemente L Vicenzini E Pantano P Raz E. Longitudinal study on low-dose aspirin versus placebo administration in silent brain infarcts: the silence study. Stroke Res Treat. (2018) 2018:7532403. 10.1155/2018/7532403 30402216 PMC6192130

[26] Ernst ME Ryan J Chowdhury EK Margolis KL Beilin LJ Reid CM. Long-term blood pressure variability and risk of cognitive decline and dementia among older adults. J Am Heart Assoc. (2021) 10:e019613. 10.1161/JAHA.120.019613 34176293 PMC8403315

[27] Zheng SL Roddick AJ. Association of aspirin use for primary prevention with cardiovascular events and bleeding events: a systematic review and meta-analysis. JAMA. (2019) 321:277–87. 10.1001/jama.2018.20578 30667501 PMC6439678

[28] Lei H Gao Q Liu SR Xu J. The benefit and safety of aspirin for primary prevention of ischemic stroke: a meta-analysis of randomized trials. Front Pharmacol. (2016) 7:440. 10.3389/fphar.2016.00440 27917124 PMC5114305

[29] Mahmoud AN Gad MM Elgendy AY Elgendy IY Bavry AA. Efficacy and safety of aspirin for primary prevention of cardiovascular events: a meta-analysis and trial sequential analysis of randomized controlled trials. Eur Heart J. (2019) 40:607–17. 10.1093/eurheartj/ehy813 30561620

[30] Marquis-Gravel G Roe MT Harrington RA Muñoz D Hernandez AF Jones WS. Revisiting the role of aspirin for the primary prevention of cardiovascular disease. Circulation. (2019) 140:1115–24. 10.1161/CIRCULATIONAHA.119.040205 31545683

[31] Berger JS Roncaglioni MC Avanzini F Pangrazzi I Tognoni G Brown DL. Aspirin for the primary prevention of cardiovascular events in women and men: a sex-specific meta-analysis of randomized controlled trials. JAMA. (2006) 295:306–13. 10.1001/jama.295.3.306 16418466

[32] Fernandez-Jimenez R Wang TJ Fuster V Blot WJ. Low-dose aspirin for primary prevention of cardiovascular disease: use patterns and impact across race and ethnicity in the southern community cohort study. J Am Heart Assoc. (2019) 8:e013404. 10.1161/JAHA.119.013404 31822218 PMC6951082

[33] Ridker PM. Should aspirin be used for primary prevention in the post-statin era? N Engl J Med. (2018) 379:1572–4. 10.1056/NEJMe1812000 30332575

[34] Diener H Hankey G. Primary and secondary prevention of ischemic stroke and cerebral hemorrhage. J Am Coll Cardiol. (2020) 75:1804–18. 10.1016/j.jacc.2019.12.072 32299593

[35] Cho L Davis M Elgendy I Epps K Lindley KJ Mehta PK. Summary of updated recommendations for primary prevention of cardiovascular disease in women: JACC state-of-the-art review. J Am Coll Cardiol. (2020) 75:2602–18. 10.1016/j.jacc.2020.03.060 32439010 PMC8328156

[36] Collins R Reith C Emberson J Armitage J Baigent C Blackwell L. Interpretation of the evidence for the efficacy and safety of statin therapy. Lancet. (2016) 388:2532–61. 10.1016/S0140-6736(16)31357-5 27616593

[37] Wright AK Kontopantelis E Emsley R Buchan I Mamas MA Sattar N. Cardiovascular risk and risk factor management in type 2 diabetes mellitus. Circulation. (2019) 139:2742–53. 10.1161/CIRCULATIONAHA.118.039100 30986362

[38] Peters SAE Muntner P Woodward M. Sex differences in the prevalence of, and trends in, cardiovascular risk factors, treatment, and control in the United States, 2001 to 2016. Circulation. (2019) 139:1025–35. 10.1161/CIRCULATIONAHA.118.035550 30779652

[39] Huebschmann AG Huxley RR Kohrt WM Zeitler P Regensteiner JG Reusch JEB. Sex differences in the burden of type 2 diabetes and cardiovascular risk across the life course. Diabetologia. (2019) 62:1761–72. 10.1007/s00125-019-4939-5 31451872 PMC7008947

